# Case report: A rare case of IgA nephropathy associated with type II Abernethy malformation

**DOI:** 10.3389/fmed.2025.1659665

**Published:** 2025-12-09

**Authors:** Wenya Cao, Xiaoli Guo, Xue Zhao, Yi Gao

**Affiliations:** Department of Nephrology, The Third Hospital of Xi’an, Affiliated Hospital of Northwest University, Xi’an, China

**Keywords:** IgA nephropathy, type II Abernethy malformation, portosystemic shunt, congenital extrahepatic shunt disconnection, case report

## Abstract

Abernethy malformation is a rare congenital vascular anomaly involving extrahepatic portosystemic shunting, with only a handful of reported cases coexisting with IgA nephropathy. We present a case of a patient who initially manifested with proteinuria and hematuria, and later developed hepatic encephalopathy, prompting vascular imaging that identified a type II Abernethy malformation characterized by an extrahepatic portocaval shunt. A diagnosis of type II Abernethy malformation was established. The patient underwent laparoscopic partial shunt disconnection, which led to a marked reduction in proteinuria (from 0.8–2.1 g/d to 0.19–0.29 g/d). This case, along with previous reports, suggests that portosystemic shunting in Abernethy malformation may be a pathogenic factor in IgA nephropathy. Surgical correction of the shunt may confer renal benefits in selected cases and deserves further investigation.

## 1 Introduction

Abernethy malformation is a rare congenital disorder characterized by abnormal development of the intrahepatic portal venous system, resulting in pathological shunting between the portal vein and systemic circulation (1). The estimated prevalence of this condition is approximately 1/50,000 to 1/30,000, making it an exceptionally uncommon disease (2). While frequently associated with various complications and congenital anomalies, renal involvement in this condition is exceptionally uncommon. We report the case of a 31-year-old male who presented with hematuria and proteinuria. During hospitalization, cirrhosis was identified. Renal biopsy confirmed Immunoglobulin A (IgA) nephropathy. Subsequent investigation of hepatic encephalopathy led to the definitive diagnosis of type II Abernethy malformation. Following surgical partial occlusion of the portosystemic shunt, the patient exhibited marked reduction in proteinuria.

## 2 Case report

A 30-year-old male was admitted on December 7, 2020, with worsening intermittent edema over 1 month following a year-long history of bilateral lower extremity edema initially noted in 2019. Hospitalization after outpatient investigations revealed significant hypoalbuminemia (serum albumin 19.3 g/L), proteinuria (2+), hematuria (3+), and 24 h urinary protein excretion of 1.18 g, leading to a provisional diagnosis of chronic glomerulonephritis with hypoalbuminemia. On admission, physical examination demonstrated mild pitting edema of the eyelids and lower extremities with unremarkable cardiopulmonary and abdominal findings. Laboratory studies confirmed proteinuria (2+) and hematuria with 64 dysmorphic RBCs/HPF, progressive hypoalbuminemia (albumin 16.0 g/L, total protein 42.3 g/L), elevated liver enzymes (AST 43 U/L), hypercholesterolemia (total cholesterol 5.93 mmol/L), markedly elevated CA-125 (142.00 U/mL) and ferritin (328.30 ng/mL), with immunology showing IgA elevation (591 mg/dL) and low C3 (53 mg/dL). Notable imaging included abdominal CT revealing cirrhosis with splenomegaly, ascites, and gastroesophageal varices, renal ultrasound demonstrating increased parenchymal echogenicity, and echocardiography diagnosing asymmetric non-obstructive hypertrophic cardiomyopathy. All other investigations—including comprehensive serological, immunological, viral, and metabolic panels—returned normal results, with no significant past medical or family history documented.

**Figure 1:**
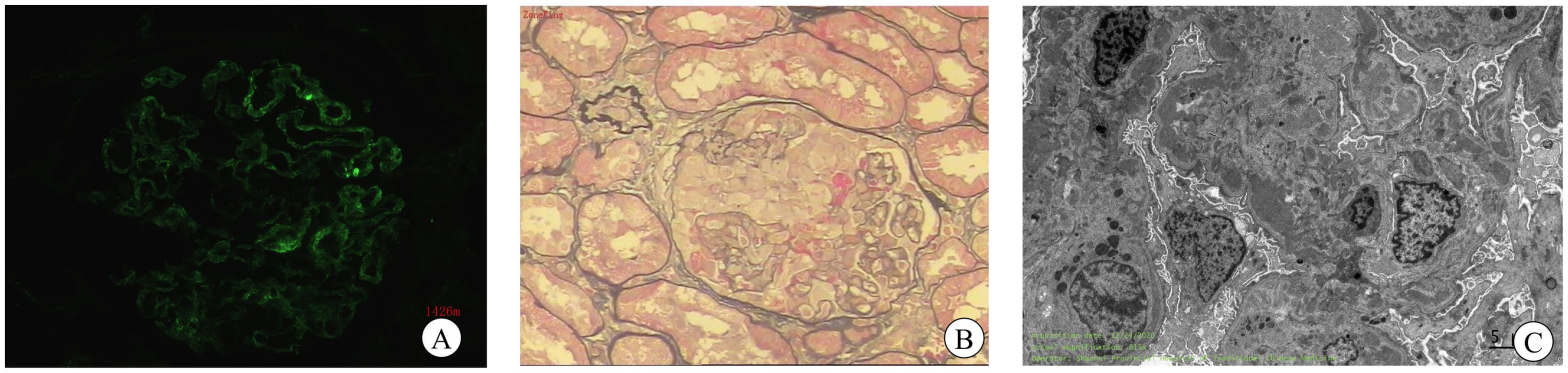
Renal histopathology. **(A)** Immunofluorescence demonstrates granular mesangial IgA deposits. **(B)** PAS stain (×400) reveals mesangial hypercellularity and matrix expansion with focal segmental sclerosis, capsular adhesion, and early crescent formation. **(C)** Electron microscopy identifies electron-dense deposits in mesangial and subendothelial zones with partial podocyte foot process effacement.

Renal biopsy performed on December 11, 2020, obtained 20 glomeruli. Light microscopy revealed three glomeruli with segmental sclerosis and capsular adhesions, one small cellular crescent, and one cellular crescent with segmental fibrinoid necrosis. The remaining glomeruli displayed mild mesangial hypercellularity and matrix expansion with focal endocapillary proliferation, alongside PAS-positive deposits in mesangial regions. Tubular findings featured vacuolar and granular degeneration with focal atrophy, while the interstitium demonstrated mild fibrosis with lymphomonocytic infiltration. Arterioles exhibited wall thickening and luminal narrowing. Immunofluorescence showed granular mesangial deposits positive for IgA (++), IgM (++), and C3 (+), with equivocal IgG (±) and negative C1q. Electron microscopy confirmed electron-dense deposits in mesangial and subendothelial zones with diffuse podocyte foot process effacement (Figure 1), establishing a diagnosis of mesangial proliferative IgA nephropathy (Lee Grade III, Oxford Classification M1E0S1T0-C1). Given negative HBV-DNA, the clinical diagnosis included IgA nephropathy, cirrhosis, and hypercholesterolemia. To mitigate bleeding risks from portal hypertension, therapy was initiated with low-dose methylprednisolone (16 mg/day), mycophenolate mofetil (0.5 g twice daily), and statin therapy. Suboptimal medication adherence led to self-guided dose reduction and treatment discontinuation, resulting in recurrent edema flares and repeat hospitalization. Proteinuria fluctuated between 0.8–2.1 g/day. Despite recommendations for angiographic evaluation and liver biopsy to determine the cause of liver dysfunction, the patient rejected invasive procedures and was managed with hepatoprotective agents. Changes in liver function parameters at baseline and during follow-up are summarized in Table 1.

**Table 1:** Liver function profiles of the patient at baseline and during follow-up.

Time (Year-Month)	TBIL (umol/L)	DBIL (umol/L)	ALT (U/L)	ALP (U/L)	γ-GT (U/L)	ALB (g/L)	PT(s)	INR	Scr (umol/L)	NH₃ (umol/L)
2020–12	17.5	4.4	36	80	26	20.3	16.4	1.27	77	/
2021–03	11.8	3.4	64	78	32	18.5	16.9	1.38	105	/
2021–11	20.1	4.7	42	119	33	17.3	/	/	85	/
2022–05	25.7	5.3	45	116	38	20.4	/	/	96	/
2023–01	26.1	5.9	77	136	47	18.6	/	/	128	/
2023–04	30.3	1.3	70	80	50	21.2	12	1.06	133	323
2023–05	17.4	5.2	62	113	72	18.4	11.2	1.11	109	108/84/69
2023–06	33.6	9.1	23	92	28	20.7	13.2	1.2	87	59
2023–09	14.3	3.9	22	53	20	18.7	15.5	1.23	119	21
2024–10	40.7	9.2	26	80	27	19.8	/	/	119	30.3
2025–03	29.9	8.2	27	89	24	19.7	12.7	1.16	168	23.2

Clinical symptoms and Child-Pugh classification							
Time (Year-Month)	WBC (*10^9/L)	RBC (*10^12/L)	PLT (*10^9/L)	Hepatic encephalopathy	Ascites	Child-Pugh classification	MELD score
2020–12	5.53	4.46	108	No	Moderate	B (9)	Low risk (8)
2021–03	7.8	3.87	79	No	Moderate	B (9)	Low risk (9)
2021–11	6.71	4.26	92	No	Mild	/	/
2022–05	5.86	4.02	84	No	Mild	/	/
2023–01	6.2	3.73	88	No	Moderate	/	/
2023–04	9.67	4.32	93	Yes (Grade 3)	Moderate	C (11)	Middle-risk (16)
2023–05	9.04	3.79	96	Yes (Grade 2)	Moderate	C (10)	Low risk (9)
2023–06	5.43	3.33	91	No	Mild	B (8)	Low risk (10)
2023–09	5.2	3.52	158	Yes (Grade 1)	Mild	B (9)	Low risk (11)
2024–10	10.5	3.67	111	No	Mild	/	/
2025–03	6.85	2.71	101	No	Mild	B (8)	Middle-risk (16)

TBIL, Total Bilirubin; DBIL, Direct Bilirubin; ALT, Alanine Aminotransferase; ALP, Alkaline Phosphatase; γ-GT, γ-Glutamyl Transferase; ALB, Albumin; PT (s), Prothrombin Time; INR, International Normalized Ratio; Scr, creatinine. The Child-Pugh classification of liver function is determined based on 5 clinical and biochemical indicators. Each indicator is graded into 3 levels (1–3 points) according to the severity of abnormality, and the total score corresponds to three liver function grades (A, B, and C). The MELD score is calculated based on three objective laboratory values and one etiology-specific factor, using the following core formula: MELD = 3.78 × ln (serum total bilirubin) + 11.2 × ln (INR) + 9.57 × ln (serum creatinine) + 6.43 × (etiology: 0 for cholestatic or alcoholic liver disease, 1 for all other causes).

**Figure 2:**
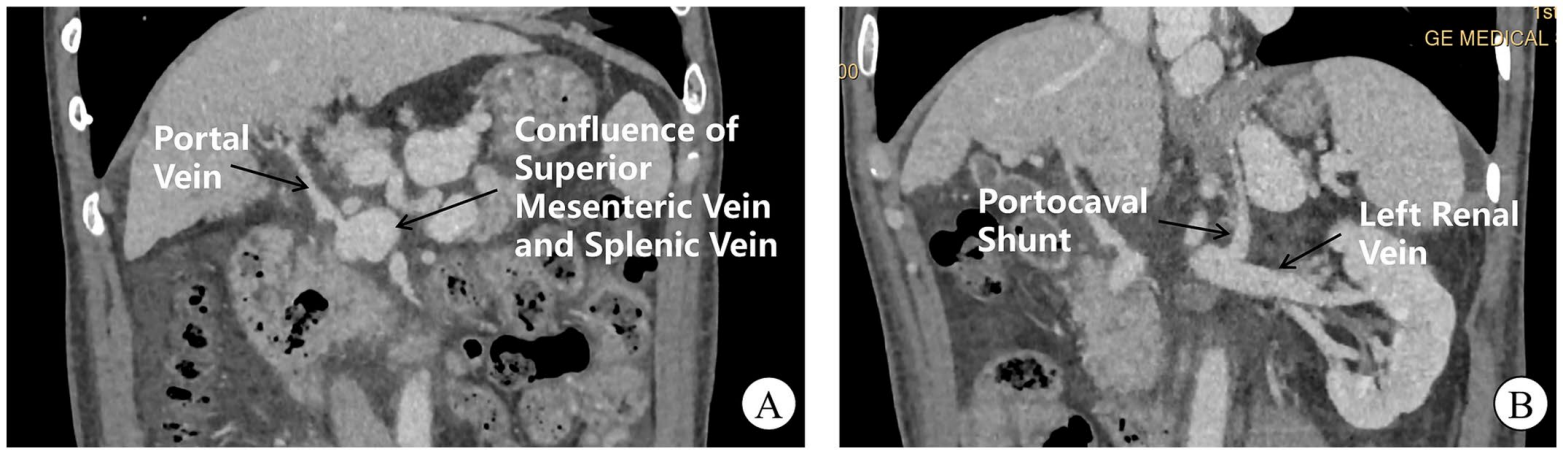
CT venography of portal and systemic circulation. **(A)** Hepatic arteriography reveals an attenuated main portal vein with a prominent aberrant shunt vessel (arrow) draining directly into the inferior vena cava at the hepatic hilum. Note dilation and tortuosity of the superior mesenteric and splenic veins converging into the shunt. **(B)** A small-caliber left subphrenic vessel (arrowhead) drains into the left renal vein.

During 2023, the patient required three hospital admissions for recurrent hepatic encephalopathy episodes. Angiography (April 2023) revealed a high-flow portocaval shunt draining into the inferior vena cava, accompanied by attenuated main portal vein branches, dilation of the superior mesenteric and splenic veins, high-risk gastroesophageal varices, and anomalous left subphrenic vessels anastomosing with the left renal vein (Figure 2). Multidisciplinary consensus confirmed type II Abernethy malformation. Preoperatively, an upper endoscopy was performed and revealed only chronic non-atrophic gastritis, without varices protruding into the lumen. In May 2023, laparoscopic partial occlusion of the portosystemic shunt was performed. The liver, inspected under laparoscopy, was small and nodular, consistent with the gross morphological diagnosis of cirrhosis. Intraoperative ultrasound confirmed the absence of focal hepatic lesions. Intraoperative exploration identified a 2.5-cm shunt vessel at the porta hepatis. Trial clamping induced marked gastrointestinal venous engorgement, prompting abandonment of complete ligation due to hemorrhage risk; consequently, 80% luminal reduction was achieved. Postoperatively, portal flow velocity increased to 10–12 cm/s (baseline: 6–8 cm/s) with progressive ammonia normalization. Notably, proteinuria decreased to 0.19–0.29 g/24 h at 2 months (pre-intervention range: 0.8–2.1 g/24 h), with urinalysis showing trace protein, 1 + hematuria, and 27.4 RBCs/HPF. Maintenance therapy with finerenone and beraprost sodium was continued. During 10-month follow-up, only one additional hepatic encephalopathy event occurred. Postoperative follow-up CT demonstrated no further progression of varices, with a marked reduction in ascites compared with preoperative findings. During subsequent follow-up, no episodes of gastrointestinal bleeding have been observed.

## 3 Discussion

Abernethy malformation is a rare congenital anomaly involving portosystemic shunts. Based on the integrity of portal vein development, it was categorized anatomically as Type I (complete shunt with absent portal vein) or Type II (partial shunt with preserved portal venous system) (1, 3). This disorder affects many systems, often leading to complications including cirrhosis, hepatic encephalopathy, and cardiovascular abnormalities (4). Notably, involvement of the renal system is uncommon. There have been less than ten reports of Abernethy malformation with glomerulonephritis. The patterns of disease include MPGN, IgA nephropathy and membranous nephropathy (Table 2) (5–10).

**Table 2:** Published cases of Abernethy malformation with glomerulonephritis.

Author(s)	Year	Abernethy subtype	Age/sex	Renal pathology	Surgery performed
David F. Schaeffer (5)	2013	Type II	18 F	IgA nephropathy	No
Wei Cunchun (6)	2017	Type II	41 M	Membranous nephropathy	No
Liu Yan (7)	2020	Type II	5 M	IgA nephropathy	Yes
Xue He (8)	2021	Type II	8 M	MPGN	No
Xin Wu (9)	2022	Type II	8 M	MPGN	No
Divya Goyal (10)	2024	Type I	11 F	MPGN	No

MPGN, Membranoproliferative glomerulonephritis; Surgery: Refers to portosystemic shunt correction procedures; Subtype classification follows Morgan & Superina criteria; Renal pathology based on kidney biopsy histology.

This patient concurrently presented with type II Abernethy malformation and biopsy-proven IgA nephropathy, progressively developing liver dysfunction, hepatic encephalopathy, and hypertrophic cardiomyopathy during the disease course. The cardiomyopathy likely represents cardiac compensatory adaptation to chronic portosystemic shunting, while hepatic dysfunction stem from portal hypoperfusion-induced parenchymal extinction (1, 11). Notably, cirrhosis itself constitutes the most common etiology of secondary IgA nephropathy, primarily mediated through aberrant IgA1 glycosylation, impaired hepatic reticuloendothelial clearance, and defective immune complex removal (12–14). In our case, the absence of prior hepatitis, insidious progression to cirrhosis/encephalopathy, and renal histopathology demonstrating dominant mesangial IgA deposits with subendothelial extension are diagnostically aligned with cirrhosis-associated glomerulopathy (15).

Critically, Abernethy malformation may instigate IgA nephropathy through portosystemic shunting prior to liver dysfunction development, suggesting an independent pathogenic role of vascular diversion in the IgA nephropathy pathogenesis (16). As demonstrated by Karashima et al., shunt fractions exceeding 90% substantially elevate nephropathy risk—likely mediated by systemic spillover of IgA immune complexes circumventing hepatic reticuloendothelial filtration, thereby triggering immune-mediated glomerular injury (17). Although our patient exhibited radiologic features consistent with cirrhosis (CT-confirmed hepatic nodularity, splenomegaly, and varices) at IgA nephropathy diagnosis, the profound and sustained proteinuria reduction following partial portosystemic shunt occlusion (from preoperative fluctuations of 0.8–2.1 g/24 h to stable 0.19–0.29 g/24 h) compellingly implicates aberrant shunting as a pivotal driver of glomerulopathy. This observation aligns with Okamoto et al.’s report of nephrotic syndrome remission after shunt embolization without immunosuppression (18). Collectively, these findings indicate that correcting portosystemic shunts directly modulates renal disease activity, operating independently or synergistically with underlying hepatic pathology.

**Figure 3:**
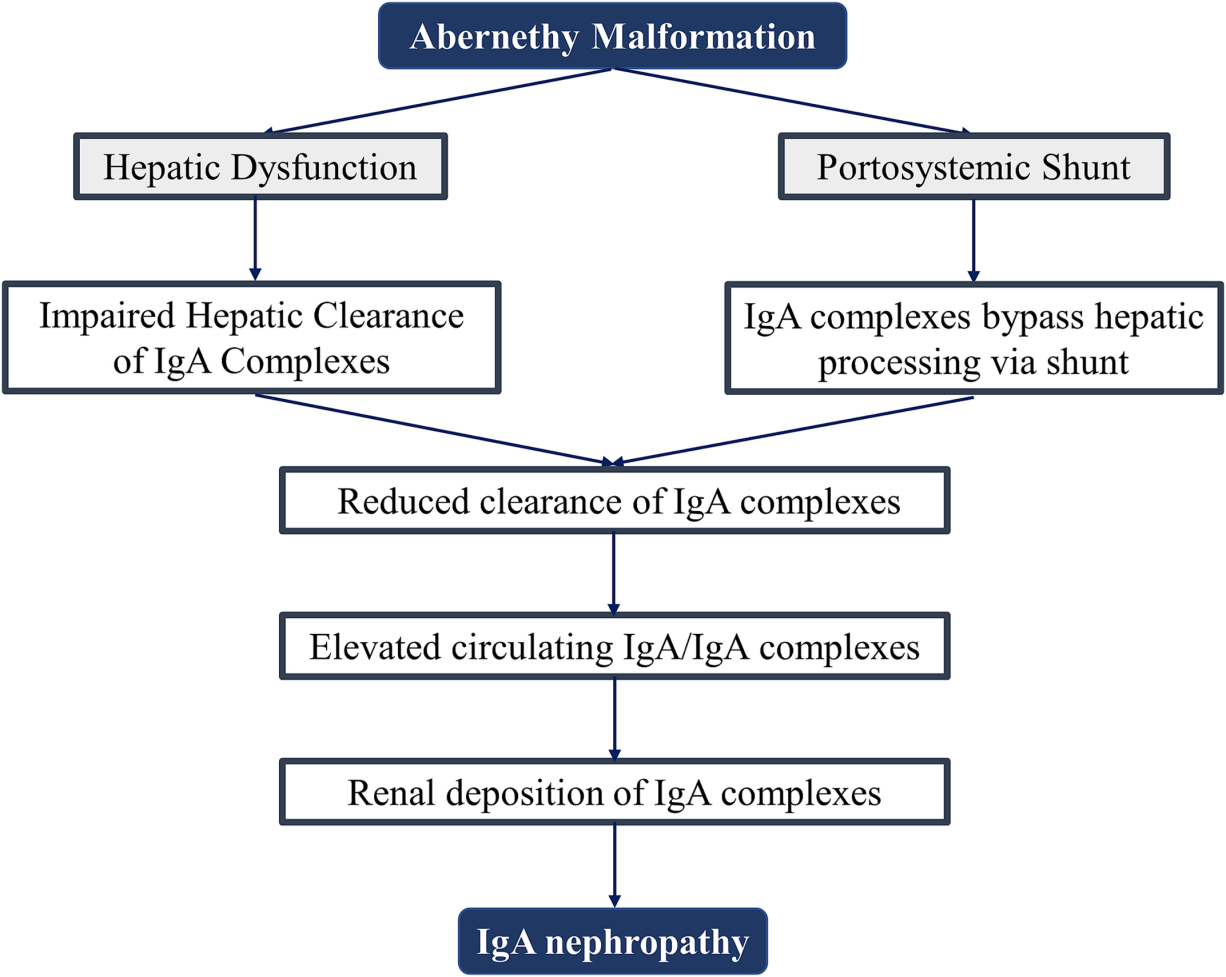
Proposed pathogenesis of IgA nephropathy in Abernethy malformation.

Furthermore, Dash et al. demonstrated that patients with non-cirrhotic portal fibrosis undergoing splenorenal shunt surgery exhibited increased IgA nephropathy incidence attributable to augmented portosystemic shunting with compromised hepatic clearance. In contrast, those with extrahepatic portal vein obstruction and preserved hepatic parenchymal function showed no post-procedural nephritis escalation (19). This implies that IgAN development is jointly governed by shunt magnitude and hepatic immunological clearance capacity (Figure 3), explaining both the absence of universal renal involvement in Abernethy malformation and the substantial age-of-onset heterogeneity.

According to presently evolved management paradigms, liver transplantation should be carried out in type I Abernethy malformation while shunt correction may be reserved for atypical type II patients symptomatic like hepatic encephalopathy patients (1). However, there is little information about the long-term benefits of portosystemic shunt treatment for kidney function. The reduction in protein and red blood cells in the urine following the procedure in our case adds evidence of a connection between these shunts and IgA nephropathy. This finding is consistent with the longitudinal study by Okamoto et al. which showed that embolization of aberrant shunts can improve nephrotic syndrome, renal function, and histological damage without immunosuppression therapy (18). Despite these encouraging signals, the strength of the claims about the long-lasting effects of shunt modification on IgA nephropathy must be shown in prospective trials.

## 4 Conclusion

Despite its rarity, Abernethy malformation necessitates multidisciplinary management due to its multisystem manifestations—particularly for monitoring long-term renal outcomes. The improvement in nephropathy symptoms following portosystemic shunt correction in this case suggests that early intervention may mitigate progressive glomerular injury. Future multi-center studies are warranted to establish the therapeutic efficacy of shunt modification on sustained renal function preservation.

## Data Availability

The original contributions presented in the study are included in the article/supplementary material, further inquiries can be directed to the corresponding author.
